# Isolation and identification of a cellulolytic *Enterobacter* from rumen of Aceh cattle

**DOI:** 10.14202/vetworld.2017.1515-1520

**Published:** 2017-12-26

**Authors:** Wenny Novita Sari, Safika, Darmawi, Yudha Fahrimal

**Affiliations:** 1 Postgraduate student of Mathematics and Applied Sciences, Syiah Kuala University, Darussalam, Banda Aceh 23111, Indonesia; 2 Department of Veterinary Infectious Diseases and Veterinary Public Health, Faculty of Veterinary Medicine, Bogor Agricultural University, Jalan Agatis IPB, Darmaga, Bogor, Indonesia; 3 Microbiology Laboratory, Faculty of Veterinary Medicine, Syiah Kuala University, Darussalam, Banda Aceh 23111, Indonesia; 4 Parasitology Laboratory, Faculty of Veterinary, Syiah Kuala University, Darussalam, Banda Aceh 23111, Indonesia

**Keywords:** Aceh cattle, cellulose, cellulolytic bacteria, *Enterobacteriaceae*, rumen

## Abstract

**Aims:**

The aim of this study was to isolate and identify a cellulolytic bacterium from the rumen fluid of Aceh’s cattle. Biodegradation by cellulolytic rumen bacteria can be used as a source of cellulolytic bacteria that act to degrade feed fibrous material so as to improve the quality of nutrients and digestibility of feed ingredients at a cheaper price than the use of commercial cellulase enzymes.

**Materials and Methods:**

Samples were collected from rumen fluid of Aceh’s cattle in Abattoirs (RPH) of Banda Aceh city, Indonesia, isolation, and screening of cellulolytic bacteria were done in Microbiology Laboratory, Faculty of Veterinary Medicine, Syiah Kuala University, Banda Aceh, Indonesia.

**Results:**

The S1 isolates showed ±2.5 cm of clear zone diameter. Microscopically, this strain was found to be a Gram-negative, *Bacillus*. Homology and phylogenetic tree analysis of 16S rRNA showed that S1 isolate has 91% of sequence similarity with that of *Enterobacter cloacae*. 91% sequence homology shown in this study proved that the S1 isolate is probably either a new species or another genus of *Enterobacteriaceae*.

**Conclusion:**

Current study suggests that cellulose hydrolytic bacteria isolated from rumen fluid of Aceh cattle on Bushnell Haas medium-carboxymethylcellulose agar, and some potent cellulose degrading bacteria have been identified.

## Introduction

Indonesia is one of the tropical countries, where ruminants are fed on lignocelluloses agricultural-products (such as cereal straw, grass, and tree foliage). Aceh cattle, one of the local ruminants germplasm in Indonesia, derived from a cross between the local cattle (*Bos sondaicus*) and *Bos indicus*. Aceh cattle are scattered in the province of Aceh and cultivated for generations as meat producers. The advantages of this Aceh cattle in addition to having a high adaptability to the bad environment are adaptation to feed which generally contains high crude fiber [1,2].

Ruminant stomach contains crude fiber such as cellulose, starch, and xylan as the basal feed for ruminants. Wahyudi *et al*. [3] have reported that the feed crude fibers were not completely converted to animal product in intensive animal farming. These materials are fermented in the rumen by microbial community including bacteria, fungi, and protozoa [4]. Rumen microbial ecosystem comprised 10^10^-10^11^ bacterial cells/mL, 10^4^-10^6^ protozoan cells/mL, and anaerobic fungi 10^3^-10^5^ zoospores/mL [5]. Furthermore, the complex has an essential role in rumen to degrade feed and supplies nutrients to host, predominantly in the form of volatile fatty acids and microbial protein [6,7].

Cellulose is one of the most important polysaccharides and the major component of plant cell walls. Bacteria also synthesize cellulose; moreover, it has greater tensile strength, versatility, and moldability. Due to its particular properties, the use of bacterial cellulose is required for different applications [8]. Cellulose is very difficult to degrade as it has homopolymer consisting of glucose units joined by ß 1-4 bond. The size of cellulose molecules (degree of polymerization) varies from 7000 to 14,000 glucose moieties per molecule in walls of plants. Cellulose has a crystalline structure, and it is surrounded by a tough lignin layer [9,10]. Rumen microbes that have been identified in the production of cellulases include bacteria, some fungi, and actinomycetes [11]. Several cellulase producing bacteria such as *Bacillus, Paenibacillus, Pseudomonas, Clostridium, Cellulomonas*, *Thermomonospora, Ruminococcus, Bacteroides, Erwinia*, *Acetivibrio, Methanobrevibacter, Gluconacetobacter*, and *Rhodobacter* species have also been identified [12-18].

Research on isolation and identification of novel cellulases producing enzymes from bacteria are still wide. In this study, a cellulolytic bacterium was isolated from rumen fluid of Aceh cattle. Biodegradation product in rumen can be used as a source of cellulolytic bacteria that act to degrade fibrous feed material so as to improve the quality of nutrients and digestibility of feed ingredients at a cheaper price than the use of commercial cellulase enzymes.

## Materials and Methods

### Ethical approval

This research was approved by the Animal Ethics Committee of Faculty of Veterinary Medicine, Syiah Kuala University (Approval No. 014/KEPH-C/III/2017). The samples used are rumen fluid waste from abattoirs (RPH) in Banda Aceh. Handling of cattle at abattoirs in accordance with good animal practices required by the Animal Ethics Committee and regulatory guidelines and local regulations.

### Sample collection

Five of Aceh cattle in which each cattle were taken 5 mL of rumen fluid were collected from Abattoirs (RPH) in Banda Aceh using a syringe. Presterilized syringe and plastic bags were used for sample collection. The samples were taken in three replications from a cattle rumen in one take-up, covering the left, right, and middle rumen parts. This aims to make the sample homogeneous.

### Isolation of cellulolytic bacteria

A sample of rumen fluid of Aceh cattle (1 mL) was suspended with 9 mL sterile double distilled water (_dd_H_2_O) (v/v) [19]. Serial dilutions from 10^−1^to 10^−6^ were prepared using sterilized _dd_H_2_O. An aliquot of 1 mL of each dilution was inoculated into Bushnell Haas medium (BHM) agar with carboxymethylcellulose (CMC) (pH 7.0) [13,20] containing (g/L) CMC (10.0), K_2_HPO_4_ (1.0), KH_2_PO_4_ (1.0), MgSO_4_.7H_2_O (0.2), NH_4_NO_3_ (1.0), FeCl_3_.6H_2_O (0.05 g), CaCl_2_ (0.02 g), and agar (20.0) [12,21] in duplicate. Plates were incubated at 39°C for 96 h.

### Screening of cellulolytic bacteria

Colonies of bacteria that grown in BHM media were exposed to Congo red 0.3% for 20 minutes, then the plates were washed with 1 M NaCl solution. In isolates indicating cellulose enzyme activity showing clear zone around the colony [13]. Colonies showing discoloration of Congo-Red were considered as positive cellulose-degrading bacteria. Furthermore, one pure colony with maximum clearing zone was isolated for further screening. The isolate was morphologically identified by Gram staining. The bacterium colony was cultured into a liquid medium and incubated for 96 h. The incubation result was centrifuged at 7000 g, the supernatant was removed, and the cell pellet was extracted for its DNA.

### DNA extraction

Total DNA was extracted separately using a commercially available kit gDNA Presto^™^ Bacteria Mini kit (Geneaid) according to the manufacturer’s instructions with slight modifications. Cell pellets were added 200 μL of extraction buffer then resuspended with pipette or vortex and then 20 μL proteinase K was added and incubated at 37°C for 30 min. During the incubation of the sample, a slow inversion was applied to the tube every 10 min. Thereafter, 200 g of buffer GB was added to the sample for the dissolution and vortex process for 10 seconds and then incubated at 70°C for 10 min. Elution buffer was heated at 70°C before its use in the next stage, and added 200 μL of absolute ethanol and lysis by a shaker. The next sample was inserted into tube column with 2 mL tube and then centrifuged 14000-16000× *g* for 2 min. The supernatant was removed and the precipitate was transferred to a new 2 mL tube.

400 μL W1 buffer was added to the column and centrifuged 14000-16000× *g* for 30 s then discarded the supernatant. 600 μL buffer wash (which has been added ethanol) is put into the column, centrifuged 14000-16000× *g* for 30 s, then discarded the supernatant, then centrifuged again 14000-16000× *g* for 3 min until the column really no supernatant, then moved to a new 1.5 mL tube. 30-50 μL elution buffer that has been heated was added right in the middle of the column carefully. It was incubated at room temperature for 3-5 min and then centrifuged 14000-16000× *g* for 1 min. Tubes containing pure DNA were stored at −20°C to prevent degradation until used for polymerase chain reaction (PCR).

### Amplification of 16S rRNA Gene

The amplification process is done using PCR method. One of the primers (BacF) used complement to sustainable regions in the bacterial and other primary domains (UniB) is based on universal sustainability of the 16S rRNA gene *Escherichia coli* [22]. The total DNA was used as a template in PCR to amplify 16S rRNA. The primers used for detecting the rumen bacteria of Aceh cattle were not a universal primer, but specific primers for bacteria [23,24]. Primer is a component of PCR that determines the accuracy of DNA sequences that want to be amplified. The sequence of primary nucleotides will be the determinant on which part of the primer will attach (anneal) to the genome. If the nucleotide sequence does not match the gene code we want, we can be sure that the resulting PCR product will be mistaken. Therefore, before stepping on the amplification stage with PCR, the primer to be used should be ascertained before its specificity.

Primers used in amplification generally consist of two types, namely, forward and reverse. The primary forward moves in the direction of the 5’-3’ DNA template strand. While reverse primers move in the direction of 3’ 5’ DNA template strand. The forward primer sequence was 5’ AGAGTTTGATC(A/C)TGGCTCAG 3’ and the reverse primer sequence was 5’ GGTTAC(G/C)TTGTTACCTGCCGGA 3’ [23], with an expected amplicon size of 1500 bp. Subsequently, 16S rRNA was amplified using the Master mix (Fermentas). A total of 25 µL of reaction mixture consisted of 10 pmol of each primer, 30 ng of template DNA, and 12.5 µL of Master mix (Fermentas). The PCR amplification was carried out for 30 cycles [25]. Each cycle consisted initial denaturing at 95°C for 5 min, 1 min 95°C, 30 s of annealing at 50°C, and 2 min of elongation at 72°C, with a final extension at 72°C for 10 min [14]. PCR product was determined by electrophoresis analysis in 1.0% agarose and 1× TAE buffer (40 mM Tris HCl, 40 mM acetate, 1.0 mM EDTA) under gel Doc (Biorad).

### Phylogenetic analysis

The sequencing results were compared using the basic local alignment search tool program on NCBI http://www.ncbi.nlm.nih.gov. and 16S rRNA gene sequence homology analysis using Genbank data. A phylogenetic tree was constructed using distance matrices by the neighbor-joining model of the MEGA 6.1 program, with substitution method maximum composite likelihood [26]. The node reproducibility for tree topology was estimated by bootstrap analysis1000 replicate data sets.

## Results

### Isolation and screening of cellulolytic bacterium

**Figure-1:**
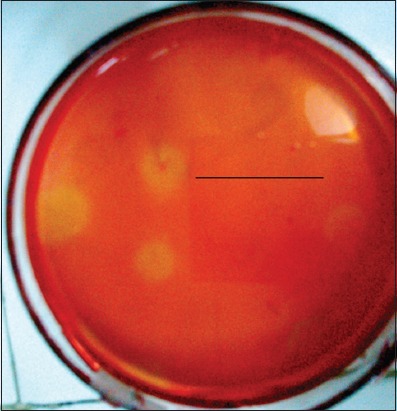
Zone of clearance on cellulose Congo Red agar plates for isolate S1 after 72 h of incubation. The formation of clearing zone around the colonies confirms the secretion of extracellular cellulase.

**Figure-2:**
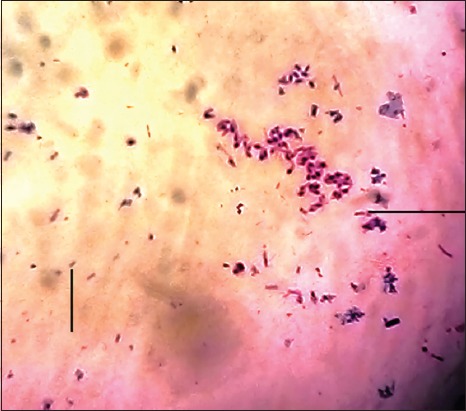
Gram staining of the cellulolytic bacterium.

Several colonies of cellulolytic bacteria were isolated from the rumen fluid of Aceh cattle on BHM-CMC agar, and cellulolytic activity quantitatively screens using Congo red staining 0.1%. The bacterium was isolated on pH 6.0-6.5 and temperature 36-39°C. Based on the calculated cellulolytic index on clear zone diameter, S1 isolate had cellulolytic activity + 2.5 cm (Figure-1). The isolate has greater cellulolytic activity than >1.0 as an indication of high cellulolytic activity [27]. Microscopically, this strain was found to be rod-shaped and Gram-negative (Figure-2).

**Figure-3:**
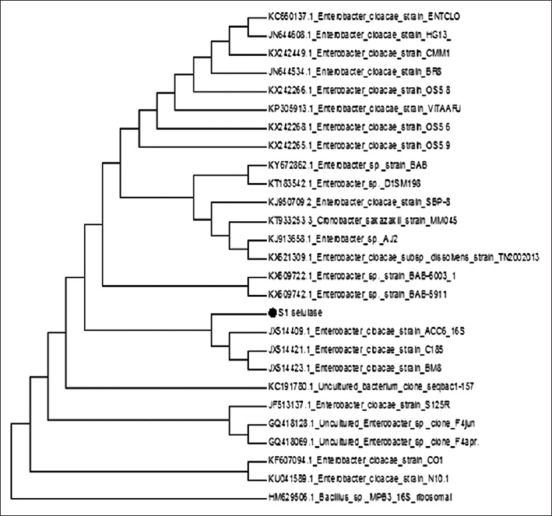
Phylogenetic dendrogram of the 16S rRNA sequence of the isolated strain.

### Analysis of 16S rRNA and Phylogenetic analyses

Homology analysis showed that isolate S1 has 91% sequence similarity with that of *Enterobacter cloacae*. The 16S rRNA sequence of S1 was kept in NCBI database with accession number MF144569. The phylogenetic tree constructed using MEGA 6.0 demonstrated that the S1 isolate is related to *E. cloacae* (Figure-3). The bacterial identification using 16S rDNA gene sequence is a widely practiced technique for microbial diversity in nature [28]. It is believed that when a sequence has a >98% similarity to a 16S rDNA of a known bacterium, it is considered to be a member of that species. However, if the percentage of homology is <97% the isolate is considered different [29]. 91% sequence homology shown in this study proved that the S1 isolate is probably either a new species or another genus of *Enterobacteriaceae*. A further study such as DNA-DNA hybridization, G-C content, and free fatty acid analysis is needed to confirm the S1 status.

## Discussion

Cellulose in the media was hydrolyzed due to a cellulolytic enzyme produced by the bacteria. The hydrolysis process produced clear zone in BHM-CMC media due to the reaction between Congo red with β-1,4-glycosidic contained in cellulose polymer. In screening for cellulase, BHM-CMC used as the substrate for endoglucanase. The activity of endoglucanase cultivated in medium indicated a relatively stable level of enzyme production during such a 3-week cultivation. A stable level of cellulolytic activity was detected also during long-term incubation [21,30].

Cellulolytic bacteria from rumen of the cattle were reported to have the optimal pH range of 6-6.5 and an optimal temperature of 37-39°C. Song *et al*. [31] reported that the cellulases from rumen bacteria retain 70% of its activity in the pH range from 5 to 7 and in a temperature range from 30°C to 50°C. pH of ruminants ranges from 5 to 7, under normal dietary conditions and the ruminal temperature can vary from 38°C to 41°C. The cellulolytic reactions of bacteria rumen have been reported to be relatively more resistant to the pH of 6.5-7 in culture.

*E. cloacae* is a Gram-negative, rod-shaped, facultative anaerobic bacterium producing biohydrogen such as formate, acetate, lactate, and ethanol. *E. cloacae* also has the ability to degrade lignin, hemicellulose, and cellulose [32-35]. Species of the *Enterobacter* are widely encountered in nature as in soil, plants but they are also pathogens and not all *Enterobacter* bacteria produce cellulase [36]. *Enterobacter* in rumen of Aceh cattle found in this study may be originated from soil, grass, straw, and foliage consumed. The bacterium has the ability to degrade cellulose contain in the feed.

Naturally, cellulase is synthesized by plants, animals, bacteria, fungi, insect, and protozoa [37]. *E. cloacae* produce cellulose three different enzymes, a complex of endo-β- 1,4-gluconase, a complex of exo-β-1,4-gluconase, and β-1,4-glucosidase [36,38]. Endoglucanase cleaves internal b-1,4-glycosidic bonds in cellulose fibers, so more free short cellulose chains. Exoglucanase hydrolyzes cellulose by release the cellobiose (b-1,4-linked glucose dimer) unit from non-reducing end of cellulose. Finally, cellobiose is hydrolyzed into two glucose monomers by b-glucosidase [39]. Hydrolysis of cellulose needs synergistic activity from various cellulases with a different specification to yield multienzyme system. The same activity is also needed to degrade cellulose molecule, product yield, and movement of cellulose chain catalytic. Cellulose multienzyme system is a microbe strategy to increase effectivity cellulase hydrolysis where each enzyme has specific function [40-42].

The microbe in the rumen can hydrolyze and ferment cellulosic polymers, so the host to get energy from the indigestible polymers. Hydrolysis of cellulose through endoglucanase and exoglucanase has been characteristic of biomass in rumen. Cellobiose, one of the major products of cellulose hydrolysis. Kajikawa and Masaki [43] reported that cellobiose intracellular hydrolysis in the rumen is considered to be more efficient than extracellular hydrolysis. Cellobiose into the cell through intracellular b-glucosidase into glucose and/or phosphorylated a derivative of glucose, which is then metabolized through central metabolism. The intracellular cellobiose requires one mole of each phosphoenolpyruvate (PEP) and ATP to produce two moles of glucose-6 phosphate. Whereas two moles of PEP are required when cellobiose is extracellularly hydrolyzed into two glucose molecules. Therefore, rumen microbial communities in ruminants are interesting sources of cellulases because these communities have adapted to utilization of lignocellulosic plant biomass.

## Conclusion

Based on the result, the cellulolytic bacterium has been successfully isolated from rumen fluid of Aceh cattle that has 91% sequence similarity of 16S rRNA with *E. cloacae* and has cellulolytic activity index ± 2.5 cm.

## Recommendations

Furthermore, this research should proceed to determine whether the isolate is as a new species through certain analysis DNA-DNA hybridization, %G+C content and free fatty acid analysis.

## Authors’ Contributions

Darmawi and Safika designed the study. WNS collected and processed the samples for isolation and screening of cellulolytic bacteria. WNS and Safika were done DNA extraction, PCR, and Phylogenetic analysis. YF and Safika interpreted the results and analyzed the data. All authors contributed equally in preparation and revision of the manuscript. All authors read and approved the final manuscript.
